# Editorial: The development of lethal prostate cancer

**DOI:** 10.3389/fcell.2023.1156392

**Published:** 2023-02-21

**Authors:** Baotong Zhang, Sifeng Qu, Xin Li, Xinpei Ci, Jiang Chang

**Affiliations:** ^1^ Department of Human Cell Biology and Genetics, School of Medicine, Southern University of Science and Technology, Shenzhen, China; ^2^ Medical Integration and Practice Center, Cheeloo College of Medicine, Shandong University, Jinan, Shandong, China; ^3^ Department of Urology, Qilu Hospital, Cheeloo College of Medicine, Shandong University, Jinan, Shandong, China; ^4^ Center for Cancer Research and Therapeutic Development and Department of Biological Sciences, Clark Atlanta University, Atlanta, GA, United States; ^5^ Princess Margaret Cancer Centre, University Health Network, Toronto, ON, Canada; ^6^ Key Laboratory for Environment and Health, Department of Health Toxicology, School of Public Health, Tongji Medical College, Huazhong University of Science and Technology, Wuhan, China

**Keywords:** prostate cancer, therapeutic resistance, NEPC, phenotypic plasticity, PSMA-based PET imaging, biopsy, tumor microenvironment (TME), natural product

Prostate cancer is the second most common cancer in men worldwide and can become lethal due to metastasis and therapeutic resistance (Sung et al., 2021). Prostate cancer is an androgen-dependent disease that relies on androgen receptor (AR) signaling for growth and survival, at least initially (Kumar et al., 2022). Therefore, androgen deprivation therapy (ADT) is the first-line therapy for prostate cancer patients with locally advanced, metastatic, and biochemically recurrent states (Auchus and Sharifi, 2020). ADT is initially highly effective, but the response to ADT wanes over time, and the disease finally progresses to castration-resistant prostate cancer (CRPC) (Ku et al., 2019). The second-generation antiandrogen therapeutics (i.e., enzalutamide) is thus developed for CRPC (Tran et al., 2009). However, the incidence of CRPC is increased following AR-directed therapy, and some metastatic CRPC further develop resistance by AR-independent mechanisms *via* phenotypic plasticity (Chan et al., 2022; Deng et al., 2022), such as the occurrence of neuroendocrine prostate cancer (NEPC) and double-negative prostate cancer (DNPC) (Bluemn et al., 2017). Therefore, it is urgent to disclose the mechanisms by which lethal prostate cancer develops (i.e., lineage plasticity) and seek powerful strategies for overcoming therapeutic resistance in advanced prostate cancer patients (Zhang et al., 2018; Quintanal-Villalonga et al., 2020). In this Research Topic, we have collected six attractive articles, including five reviews and one original research, that discuss lethal prostate cancer, from basic molecular mechanistic details to clinical diagnosis and therapies.

As an advanced stage of prostate cancer, NEPC develops therapeutic resistance and confers a poor prognosis (Liu et al., 2022). Understanding the crucial contributors in NEPC formation helps better develop therapeutic strategies for this lethal form of prostate cancer (Wang et al., 2021; Cheng et al., 2022). Two review articles submitted to this Research Topic systematically summarized the intrinsic and extrinsic mechanisms underlying NEPC development, respectively. Sreekumar and Saini (fcell-11-1075707) elucidate the critical roles of transcription factors and chromatin modifiers in NEPC reprogramming. Notably, the authors discussed a fascinating question of whether AR remains active in NEPC, and they suggest that 50% of treatment-resistant prostate cancer with neuroendocrine features retain nuclear AR, but the downstream transcriptional networks are reprogrammed from the canonical pattern to neuroendocrine-like pattern by lineage modulators such as EZH2 and KLF5. Importantly, the authors summarized the preclinical evaluation and clinical trials of transcription factors and chromatin modifiers in NEPC, providing valuable information for translational purposes. The second review article from Zhou et al. (fcell-10-955669) reviewed the unique niche of NEPC. They suggested that both the cellular and the non-cellular components of the tumor microenvironment (TME) induce or modulate NEPC formation. Impressively, they discussed different cellular components in the microenvironment of NEPC, including tumor-associated macrophages (TAMs), myeloid-derived suppressor cells (MDSCs), cancer-associated fibroblasts (CAFs), mesenchymal stem cells (MSCs) and vascular endothelial cells (VECs). In addition, they summarized which and how paracrine signalings are employed for recruiting and activating cellular components. TME enhances prostate cancer cell stemness, induces cell plasticity, and is thus required for NEPC differentiation. Therefore, interference of TME crosstalk could provide new directions for clinical NEPC treatment.

After understanding the molecular and cellular mechanisms underlying advanced prostate cancer development, our Research Topic also highlights novel diagnostic strategies to monitor prostate cancer progression, providing promising tools for directing proper therapies. Prostate biopsy is a critical factor affecting prostate cancer detection (Connor et al., 2023). Improving prostate biopsy accuracy is thus in great demand. Since multiparametric magnetic resonance imaging (mpMRI) is widely applied, it provides valuable information and helps to identify the target area during a biopsy. Two biopsy methods have different biopsy puncture paths (i.e., transperineal vs transrectal), and each contains advantages and limitations. By using mpMRI, Jiang et al. (fcell-10-851359) reported a personalized prostate biopsy pattern. The suspected lesion site was determined by mpMRI examination before biopsy, while the biopsy approach (transperineal or transrectal) was selected individually according to the lesion site. The personalized prostate biopsy can improve prostate cancer detection rate, which shows advantages to traditional transrectal prostate biopsy. These findings provide valuable insights that mpMRI is necessary before biopsy, and personalized biopsy can improve accuracy.

Focusing on another appealing diagnostic improvement, a review article by Chen et al. (fcell-10-958180) systematically reviewed prostate specific membrane antigen (PSMA)-based positron emission tomography (PET) scan. Conventional imaging modalities, such as magnetic resonance imaging (MRI), abdominopelvic computed tomography (CT), and whole-body bone scans (BS), are recommended as stand imaging techniques but lack sufficient accuracy. PSMA-based PET imaging can accurately detect local and disseminative lesions and describe their molecular features quantitatively in CRPC patients. Therefore, PSMA-based PET would help in detecting metastasis and monitoring treatment responses. Moreover, PSMA-based PET imaging could be combined with PSMA radioligand therapy (RLT) for better options in CRPC treatment.

Our Research Topic also shed light on novel therapeutic strategies for prostate cancer patients. The review article from Singla et al. (fcell-09-745177) focused on natural products for the management of CRPC. The authors collected evidence to support that numerous natural products are effective for CRPC treatment potentially. However, the efficacy of these natural compounds is limited due to hydrophobic molecules, instability, low pharmacokinetic profile, poor water solubility, and high excretion rate. A promising way to weaken these unfavorable pharmacological properties is *via* nanoparticle formulations, which improve pharmacokinetic drug profile and transportation. The combination of natural compounds and nanoparticles is promising but still under testing.

In addition, our Research Topic includes a meta-study from Xie et al. (fcell-09-792597) to analyze the side effects of radiotherapy for prostate cancer. Radiotherapy mainly consists of external beam radiotherapy (EBRT) and brachytherapy (BT) (Aboagye et al., 2023). There are two major considerations when making the optimum treatment option for prostate cancer, tumor control probability (TCP) and quality of life (QOL). Sexual function is a major factor to affect patients’ QOL (Martin et al., 2022). The authors investigated a total of 2340 prostate cancer patients who received either EBRT or BT. They found that BT showed advantages in maintaining sexual function in localized prostate cancer patients during the immediate post-treatment period.

The articles included in this Research Topic are particularly interesting because they systematically summarized recent mechanistic findings underlying NEPC, a lethal form of prostate cancer, and highlighted novel diagnostic and therapeutic methods for advanced prostate cancer patients. These efforts will improve understanding and lead to potential clinical applications for cancer treatment.
